# Practicing Sport in Cold Environments: Practical Recommendations to Improve Sport Performance and Reduce Negative Health Outcomes

**DOI:** 10.3390/ijerph18189700

**Published:** 2021-09-15

**Authors:** Hannes Gatterer, Tobias Dünnwald, Rachel Turner, Robert Csapo, Wolfgang Schobersberger, Martin Burtscher, Martin Faulhaber, Michael D. Kennedy

**Affiliations:** 1 Institute of Mountain Emergency Medicine, Eurac Research, 39100 Bolzano, Italy; rachel.turner@eurac.edu; 2 Institute for Sports Medicine, Alpine Medicine and Health Tourism (ISAG), UMIT, Private University for Health Sciences, Medical Informatics and Technology, 6060 Hall i.T., Tirol, Austria and Tirol-Kliniken GmbH, 6020 Innsbruck, Austria; tobias.duennwald@umit.at (T.D.); wolfgang.schobersberger@tirol-kliniken.at (W.S.); 3 Centre for Sport Science and University Sports, University of Vienna, 1010 Vienna, Austria; robert.csapo@univie.ac.at; 4 Austrian Society for Alpine and High-Altitude Medicine, 6414 Mieming, Austria; martin.burtscher@uibk.ac.at (M.B.); martin.faulhaber@uibk.ac.at (M.F.); 5 Department of Sport Science, University Innsbruck, 6020 Innsbruck, Austria; 6 Athlete Health Lab, Faculty of Kinesiology, Sport, and Recreation, University of Alberta, Edmonton, AB T6G 2R3, Canada; kennedy@ualberta.ca

**Keywords:** cold exposure, health, exercise performance, athlete, warm-ups

## Abstract

Although not a barrier to perform sport, cold weather environments (low ambient temperature, high wind speeds, and increased precipitation, i.e., rain/water/snow) may influence sport performance. Despite the obvious requirement for practical recommendations and guidelines to better facilitate training and competition in such cold environments, the current scientific evidence-base is lacking. Nonetheless, this review summarizes the current available knowledge specifically related to the physiological impact of cold exposure, in an attempt to provide practitioners and coaches alike with practical recommendations to minimize any potential negative performance effects, mitigate health issues, and best optimize athlete preparation across various sporting disciplines. Herein, the review is split into sections which explore some of the key physiological effects of cold exposure on performance (i.e., endurance exercise capacity and explosive athletic power), potential health issues (short-term and long-term), and what is currently known with regard to best preparation or mitigation strategies considered to negate the potential negative effects of cold on performance. Specific focus is given to “winter” sports that are usually completed in cold environments and practical recommendations for physical preparation.

## 1. Introduction

Athletes who participate in winter sport or practice sport in specific areas of the world often face cold environmental conditions. Despite the fact that humans operate within a narrow limit of optimal core body temperature to ensure thermal homeostasis, cold exposure is not by itself considered a barrier to perform sport [1]. In fact, humans are well equipped with extensive thermoregulatory mechanisms designed to facilitate adaptability to large ranges in environmental temperature [2], especially cold environments [3]. Yet, cold or what is considered “cold” in the context of human interactions with the environment is not well defined. Previous initial research defined the “thermal neutral zone” as “the maximum gradient (Skin Temperature—Air Temperature) over which the body can maintain its temperature without an increase in heat production” [4]. This means that at ambient air temperatures less than 28.5 °C, defined as the “lower critical temperature”, the body responds with a number of coping mechanisms to maintain body temperature. Thus, some would consider air temperatures less than 28.5 °C as cold in the strictest sense of human thermoregulation physiology. However, energy expenditure, subcutaneous fat thickness, and clothing are all known to extend this lower critical temperature where the body does not perceive cold as a physiological stressor affecting thermoregulation. For instance, an air temperature of 5 °C may be considered as cold. However, an exercising, heavily clothed person in that environment may be hot [5]. Thus, for the purposes of this paper, “cold” is considered as a physiological stressor where the ambient environment may impair exercise/sport performance. In addition, circumstances combined with cold, e.g., rain and wind, can create life-threating conditions due to a fatal drop in body core temperature. This unfortunately is described in several reports where athletes died due to hypothermia during sport competitions, where a drop in ambient air temperature contributed to the fatalities [6,7].

Decades of research have focused on the pathophysiological impact of cold stress and potential cold injury in isolation. In contrast, relatively little attention has been paid to how we may better provide evidence-based preparation and support strategies to combat excessive environmental cold stressors and enhance exercise performance in these environments. Thus, this review is not intended to describe freezing-cold injuries or associated pathophysiological conditions (e.g., hypothermia, frostbite), nor is it intended to systematically review the literature or to specifically describe physiological responses related only to cold exposure, for which the interested reader is referred to [1]. Rather, this review provides a review of pertinent literature which summarizes research findings dealing with the effects of cold exposure on exercise performance in different types of exercise (i.e., endurance, strength), describes potential health issues (short- and long-term) with special focus on practical applications and recommendations. Providing evidence-based recommendations is of great importance, yet due to the complexity of the topic it is difficult to achieve. It is the position of the American College of Sports Medicine that exercise can be performed safely in most cold weather environments, without incurring cold weather injuries, as long as a comprehensive risk management strategy is employed [8]. This includes (a) careful identification/assessment of the cold hazard in different populations and those factors thought to contribute to cold weather injuries, (b) development and formal implementation of controls intended to mitigate cold stress/strain, and (c) utilization of administrative oversight to ensure controls are enforced or modified as required. As a result, in some sports, such recommendations or “cold exposure limits” are outlined in the competition rules. For instance, the International Ski Federation (FIS) states the following for cross-country skiing: “There are three main factors to be considered by the Jury regarding cold weather safety: the temperature; the duration of the exposure, and the clothing and other protection against cold weather. These factors together with any other relevant information such as the “wind chill factor” must be taken into consideration when a decision is made regarding cold weather.” Furthermore, it is stated ”If the temperature is below −20 °C, measured at the coldest point of the course, a competition will be postponed or cancelled by the Jury. With difficult weather conditions (e.g., strong wind, high air humidity, heavy snowfall, or high temperature) the Jury may, in consultation with the Team Captains of the participating teams and the Chief of medical and rescue service responsible for the competition, postpone or cancel the competition”. (https://assets.fis-ski.com/image/upload/v1624284540/fis-prod/assets/ICR_CrossCountry_2022_clean.pdf, accessed on 20 August 2021). The International Biathlon Union (IBU) follows similar guidelines (https://res.cloudinary.com/deltatre-spa-ibu/image/upload/nuzknw5thqfxq25jimm4.pdf, accessed on 20 August 2021). In other sports (e.g., alpine skiing, where athletes tape their face to protect from cold, or soccer), no cold thresholds are defined. Yet in a document of the Union of European Football Association (UEFA), the following is mentioned for soccer: “If temperature is −15 °C or colder, the match is postponed unless both teams agree to play” (https://circabc.europa.eu/sd/a/ac474c63-fd9d-485d-acc8-593fbf7fd232/Foot-20180927-WG-pres%20EUFA.pdf, accessed on 20 August 2021).

Consequently, as mentioned, recommendations are paramount for both safe competitions and good sport performance, yet scientific evidence to support adequate thresholds is somewhat scarce and more research in this regard is warranted. Nonetheless, this review represents an attempt to provide practitioners, coaches, and personnel working with athletes with some information on potential performance effects, health issues, and how best to advise athletes of various sport disciplines to prepare for and behave when training, competing or exercising in the cold.

## 2. Effects of Cold Exposure on (Sport) Performance

Sport performance depends on a variety of factors such as the capability of the cardiovascular system to deliver oxygen to the working muscle, the ability of the metabolism to produce a sufficient amount of energy mostly from stored substrates, the adequate function of the neuromuscular system, and maintained psychological capabilities (e.g., cognitive function, motivation, pain endurance). Cold exposure may affect these factors as reviewed in detail elsewhere [1,9,10], thus influencing sports performance. It needs to be mentioned that when investigating the effects of cold exposure on exercise performance and physiological responses, cold environments in the range of −20–15 °C have been mostly compared with temperate (around 20 °C) and/or warm conditions [1].

Given that muscle temperature and neuromuscular function are linearly related [11], it stands to reason that the maintenance of muscle temperature is paramount to performance in cold environments. In fact, the maximal contractile force of skeletal muscles is decreased by cooling [12] and force velocity curves are shifted left [13], implying that cold stress may lower the range of force production and potentially slow movement velocity. Translated to endurance performance in the cold, this means participants are likely operating at a greater percentage of their maximal voluntary force production and might not produce the same velocity of movement in the power phase of a movement (such as double poling in cross country skiing). The influence of cold stress on neuromuscular function occurs in both sports that require water immersion and terrestrial-based endurance sports, although endurance athletes can tolerate significantly colder air temperatures in terrestrial sports (safe minimum temperatures is −15 °C in cross country skiing) [14] compared with water-based sports such as ultra-endurance swimming, where the safe minimum temperature is 14 °C [15]. The heat conductance/dissipation capacity of water is approximately 25 fold greater than that of air [16]. Therefore, a water environment can cool the body much more effectively than air, rapidly surpassing the innate protective mechanisms against such cooling, i.e., peripheral vasoconstriction and increased metabolic heat production [16].

In addition, the perceptual responses to cold in sporting environments is not well studied. Borrowing from standardized exercise protocols where adequate clothing was allowed, the rating of perceived exertion (RPE) is not different in extreme cold running (<−15 °C) compared with 0 °C [17]. Additional evidence points to the protective effect that clothing can have on the perceptual effects of cold exercise environments, where high permeability clothing increases the “rating of cold perception” compared with “low permeability clothing” during walking exercise [18]. Furthermore, RPE during exercise is the same in conditions where skin temperature is “warmed from cold” or “cooled from warm” (skin temperature range: 29 to 34 °C) [19]. Thus, RPE is probably not affected by climatic conditions; however, research which has examined thermal comfort has found that ambient environment does affect perceptions of comfort. This research into human thermo behaviour illustrates that humans use a variety of choices to maintain body temperature, including the addition and removal of clothing [19]. Thermo behaviours are driven by ambient, skin, and core temperature, which either ensure maintenance of thermal comfort or a reduced thermal discomfort [19]. In the context of cold weather exercise, as previously indicated, metabolic heat production reduces the need to wear as much protective clothing, given the maintenance of core temperature in cool (<20 °C) and colder (−20 °C) environments [5,20,21]. It is probable, given the current research on thermo behavior, that athletes who train and compete in the cold abide by two behaviours to regulate thermal comfort. Firstly, athletes likely wear less clothes, understanding that being “cold-uncomfortable” in the early stages of a workout is acceptable, knowing that metabolic heat production will provide them with a feeling of “warm-comfortable” once the workout progresses. Secondly, experienced cold weather athletes understand that metabolic heat production can lead to sweating, which will alter thermal comfort [22]. Therefore, they preferentially choose protective clothing that is highly breathable and has wickable properties that offer some insulative value while exercising. The reasons for their choice in clothing is mostly to reduce the chances of sweating, which can alter clothing insulative value, increase skin clamminess, and create cooling due to sweating [18], ultimately altering thermal comfort [22]. It is also likely that less-experienced athletes might opt to overdress, in comparison with more experienced athletes, to maintain initial thermal comfort at the start of a workout. However, as of yet, this clothing preference has not been studied to our knowledge. Furthermore, over the course of the cold portion of a training year (i.e., fall and winter), cold weather athletes might feel more comfortable at colder temperatures, which could improve both focus on exercise effort and intensity, although this has also not been studied extensively. It is notable that wind exposure on facial skin has an accelerating effect on both physiological responses and thermal sensations in cold ambient air at rest [23] and during exercise [24]. Given the fact that cold weather athletes are likely to have exposed skin on their face [25], it should be noted that covering of the cheeks, forehead, nose, and neck might improve regulation of thermal comfort, especially in windy conditions greater than 5 m/s at temperature ≤−10 °C [23]. Finally, it has been shown that unclothed females during running exercise operate at about a 2 °C less skin temperature than males, with the same thermal sensations [26]. This finding implies that female athletes might be more prone to muscle temperature decreases under the same thermal conditions, although more research is needed to further elucidate how this difference may affect skin and muscle temperature in a clothed condition, and how this subsequently effects exercise/sport performance.

From a metabolic standpoint, acute cold stress increases oxygen consumption in a dose-dependent manner in both active and passive cold water immersion [15,16] and cold air terrestrial sports [1]. Furthermore, previous research has shown that habitual exposure to colder environments found in the winter (ambient temperatures of 5 °C) results in greater metabolic heat production in response to mild acute cold exposure at rest [27]. However, it is understood that the metabolic response to acute cold exposure is individual [28] and based on ethnicity [29], which has implications for individualized athlete management of cold stress in sport. During exercise, cold stress has been shown to decrease submaximal oxygen consumption (VO_2_) cost, where increased respiratory exchange ratio (RER) at moderate sub-zero temperatures between 0 and −10 °C reflects a shift to carbohydrate metabolism compared with 10 to 20 °C, when core temperature is maintained [30]. In extreme cold conditions such as −20 °C, similar VO_2_ cost and cardiovascular responses have been found compared with 20 °C [31]. Conversely, when shivering, thermogenesis is induced during sub-maximal exercise, then VO_2_ consumption is increased in comparison with non-shivering conditions [32,33], where shivering at rest can increase VO_2_ to as high as 1.5 L/min [34]. How cold stress affects VO_2max_ is equivocal where VO_2max_ is reduced in severe cold [35] or is not changed in less severe conditions of −14 to −9 °C [36]. Additional negative effects of cold air exposure while performing endurance sports include decreased visual acuity, general alertness, and reflexes [37]. It is implied that the consequences of decreased acuity increase mistakes in team-based sports such as rugby, leading to increased risk of injury [37]. However, how cold exposure affects reflexes, dexterity, decision making, and general alertness in sport is not well known. Furthermore, most human cold environment research studied acute exposure to cold environments, whereas cold acclimation studies are relatively understudied relative to altitude or heat acclimation studies [1]. Repeated short-term cold immersion studies using water immersion have been shown to have a prolonged effect [38], while “mild cold exposure” can lead to “non shivering thermogenesis” and less pain and discomfort when exposed to acute cold conditions [29]. As the authors suggest, cold acclimation can also benefit cognitive focus, a distinct advantage in sport environments [29]. In summary, it is likely that those living and training in cold weather environments have an advantage relative to warm climate athletes competing in cold environments. Practically, winter sport nations all have a prolonged winter season, where athletes from those countries all might benefit from cold acclimation leading into a competition. However, there may be instances in team sports performed outside, such as soccer or individual events such as cross country running, where an unseasonably cold environment would advantage cold weather countries relative to warm weather countries. Investigating the long-term exposure of cold on exercise performance and the advantages that cold habituated competitors have relative to warm weather competitors forced to compete in the cold would be an important future research focus.

More specific information on the effects of cold during endurance and strength exercise will be briefly described. Where appropriate, additional recommendations for practicing sport in the cold are given for the different types of respective exercise and sports.

### 2.1. Effect of Cold on Endurance Exercise and Sport

Endurance exercise can be defined as a structured endurance activity that is performed for the purposes of improving physical health and fitness [39]. This type of exercise is characterized as continuous aerobic steady state exercise, where the related training is focused on improvement of an individual’s ability to intake, transport and utilize oxygen for function, which in turn improves VO_2_max [40]. Additional benefits include an individual’s ability to perform submaximal muscular contractions repetitively [41] at intensities which solicit aerobic metabolism primarily [40]. Given that endurance exercise bouts or events are completed over durations ranging from 75 s [41] to beyond 24 h [42], the influence of environment plays a key role in endurance performance [43,44]. For those endurance sports or activities that are completed in the winter or environments where cold exposure is significant, air temperature, wind speed, and wetness should be considered [45]. Ultimately, the success of an individual to maintain a required intensity in a specific mode of exercise in cold environments is a balance of cold exposure and metabolic heat production, where inability to maintain thermoregulation will affect exercise performance and health [6,45]. The following sections explore some of the key effects of cold exposure on endurance exercise and what is known in specific sports that are completed in cold environments. For the purposes of this review, we will focus on ambient environments where cold stress is a limiter of endurance performance rather than an aid to performance.

#### 2.1.1. Dose-Dependent Nature of Cold Exposure on Endurance Exercise

Endurance sports have a minimum and maximum temperature recommendation to ensure safety of participants [45]. For cold weather sports, the minimum value is more important given the potent effect cold stress can have on performance [34]. For example in endurance sports where cold is a factor, competition temperature minimums of 16 °C for open water swimming [15] and −20 °C for cross country skiing exist [14]. However, these minimum temperatures for competition are rooted in the safety of participants [14,15]; these minimums highlight the inverted U shape that environmental cold exposure can have on endurance performance [20]. To elaborate, in summer sports where heat stress is a key factor in endurance performance, hyperthermia may impair performance, whereas cooler temperatures that are below 20 °C likely benefit performance [21]. In fact, endurance performance is optimized at ambient air temperatures of approximately 10–13 °C [20,46,47], in comparison with temperatures greater than 25 °C [48]. Thus, in the inverted U relationship between ambient temperature and exercise performance, the ascending right portion of the U would end at an apex of 10–13 °C. With lower temperatures which are low enough to significantly lower muscle temperature, cold becomes detrimental to endurance exercise [20]. This would be the descending left portion of the inverted U, where sub-zero ambient temperatures will start to decrease performance. In summary, endurance exercise is likely optimized in cool environments ≤20 °C; however, as external cold stressors (air, wetness, wind speed) overwhelm metabolic heat production to maintain body temperature and muscle temperature, endurance performance can be negatively affected [1].

#### 2.1.2. Influence of Cold on Endurance Sport Performance

The effect of cold on endurance performance, where performance is defined as an external measure of time to fatigue, finish time, or velocity, is clear. Given that cool temperatures in the range of 10 °C enhance exercise performance, we will examine ambient air temperatures <10 °C [20,21] on endurance performance. To exemplify how potent cold air can be in specific sports, it has been reported that −18 to −28 °C conditions in a multiday cross country ski race resulted in only 6% of participants finishing due to extreme discomfort and fatigue [49]. Interestingly, independent of cold exposure, the high energy cost of moving on snow with a high friction coefficient due to cold also alters metabolic demands in endurance sports such as cross country skiing [50] or ski mountaineering [51]. Similar performance decrements exist in endurance swimming performance, where duration is significantly reduced if body core temperature is not maintained [52]. Other known effects of cold on performance in winter sports include cold induced tremors, which can affect shooting acuity in biathlon, thereby increasing race finish time via penalty loops or time penalties [53].

In the above examples of performance decrements due to cold, the key suggestion made by researchers to improve performance is to wear more protective or insulative clothing. As cited by others [54], clothing is the most modifiable factor which can reduce cold induced environmental risk affecting performance [55]. The use of inadequate clothing has been established in male cross country skiers who wear thin racing suits instead of traditional winter clothing [36]; however, the influence of cold on female endurance athletes wearing similar clothing is negligible down to −14 °C [56]. This corresponds with previous studies, which found that short duration, intense exercise performance in females is not compromised if they wear insulative high performance clothing even at −20 °C [17]. This highlights a future area of endurance performance research in cold weather environments, where female tolerance to cold might be better than similarly fit males. When examining other factors which might improve endurance performance in the cold, acclimation is a viable approach to improved performance and has been reviewed elsewhere extensively [57,58]. Specific instances where acclimation may improve endurance performance would be marathon running where sudden inclement weather such as rain [59] affects running performance relative to optimal marathon conditions [60]. In addition, multi-day ski traverses in polar regions of the world have shown to be a “living lab”, where prolonged exposure to extreme cold alters performance and physiological tolerance to cold weather [58,61]. Finally, higher body fat enables open water swimmers to stay in the water for a longer period of time, which can improve the success of ultra-endurance open water swims [62]. In terrestrial sports, however, where economy of motion is affected by body mass, increased body fat to improve insulative protection to cold would not be considered effective [63]. Thus, a focus on protective and insulative clothing that allows wicking but maintains skin and muscle temperature should be explored in future cold weather endurance sports research.

### 2.2. Effect of Cold on Strength Exercise and Balance

Besides the established effects of cooling applications such as cold-water immersion or icing procedures that have previously been implemented to reduce local and core temperature of the body, only a few studies describe the impact of cold ambient air exposures on muscular strength [1,64]. However, in “real-world” scenarios, apart from swimming, exercise is frequently performed in cold environments and resting periods may precede the activity. In this regard, cold ambient air exposures may be more related to real exercise conditions.

Maximal wrist flexion force contraction following repeated dynamic eccentric-concentric exercise bouts (6 × 20 min) performed at an air temperature of 5 °C was reported to decrease more after lowering of mean skin temperature (i.e., systemic cooling) compared with local cooling (by 26% and 17%, respectively) [65]. In healthy sedentary subjects, significant impairments in drop jump performance were observed after a 60 min passive exposure in a climate chamber at varying temperatures (20 °C, 15 °C, and 10 °C) [66]. Notably, the performance decrement already appeared at relatively mild cooling of 20 °C and the extent depended from the degree of cooling [66]. Comparable magnitude of performance reduction was found in older woman (mean age 78 years) exposed for 45 min to an ambient temperature of 15 °C, with significant decreases in leg extensor power when compared with temperate conditions of 25 °C [67].

In order to evaluate the effects of cold on muscular strength implementing a more praxis-oriented approach, lightly clothed recreational athletes were exposed to a natural cold environment, where they underwent specific athletic performance tests [68]. Interestingly, after passive exposure of only 15 min at 6.1 °C, vertical jump height and agility performance significantly decreased when compared with the control condition (17.2 °C) [68]. As sprint performance remained unaffected from the fall in temperature, authors speculated that muscle coordination may be more affected from acute exposure to cold ambient temperatures than contraction velocity or power, and that coordination decrements may be more obvious during exercises with higher complexity such as jump performance or agility [68].

In another study, effects of cold (−14 °C) and cool temperatures (6 °C) on double poling (DP) performance using clothing concepts realistic to cross-country sprint skiing were evaluated [69]. Peak power output during the 30 s and the 2 min DP sprint tasks decreased more in the cold condition [69]. In addition, after the exposure to cold air for 54 min (including repeated maximal exercise bouts) an incremental test to exhaustion was performed, resembling true cold stress experienced by the athletes. Similarly, in this test peak power output was significantly more reduced at −14 °C [69].

Cold stress may also alter manual performance (e.g., grip strength), depending on the type of cooling and performance tasks [70]. This can be relevant to climbing sports, which are frequently performed outdoor under varying temperatures. When the effects of cold air (10 °C vs. 24 °C) were tested on climbing-specific tasks in lightly dressed subjects (T-shirt and shorts), maximum voluntary contraction (MVC) strength of the finger flexor muscles was not impaired by temperature, whereas time to task failure was even improved [71]. In addition, no effect on force variability was observed during the fatiguing task at 40% MVC [71]. Similarly, isometric force control during submaximal hand grip tests at 30% and 10% of MVC following mild whole-body cooling at 8 °C (i.e., decreased mean skin temperature without fall in rectal temperature) did not differ between cold and control temperature (25 °C) [72]. Hand grip MVC decreased over time without an effect of temperature on MVC measured in the end of the 78 min cold exposure [72].

Studies mentioned above give important insight into the effects of cold on strength in more realistic life situations. However, a common condition when exercise is performed outdoors can be the combination of different environmental stressors. Many strength-related outdoor sports such as skiing, climbing, ski mountaineering or cross-country skiing may be performed at altitude, exposing an athlete concurrently to cold and hypoxia. In this regard, the effects of combined exposure to altitude (FiO_2_: 0.13) and cold (5 °C) on forearm muscle fatigue were evaluated [73]. Repeated low-resistance exercise during exposure (70min) led to a reduction in MVC of finger flexors by 8.1% in hypoxia and 13.9% in the cold [73]. Interestingly, the combination of hypoxia and cold revealed an absolute additive effect showing decreased MVC force output by 21.4% when compared with thermoneutral normoxia (22 °C) [73]. Likewise, an additive relative effect reducing time to exhaustion during high-intensity dynamic knee extension was found during combined exposure to cold and hypoxia under similar conditions [74]. This reduction in time to exhaustion is likely related to crosstalk between the highly integrative, overlapping mechanisms which control heat conservation, preferential oxygen delivery and blood pressure maintenance in cold, hypoxic conditions even evident at rest [10]. For further information on the combined effects of cold exposure and hypoxia, the interested reader is referred to Mugele et al. (2021) [10]

In addition to force generation, exposure to cold environments and cooling of large parts of the lower extremities may also negatively affect postural control and dynamic balance [75,76]. Such detrimental effects may still be apparent when exposures to cold air are repeatedly performed [75]. The temperature-sensitive firing patterns of exteroceptive mechanoreceptors [77] as well as increases in joint and muscle viscosity [78] may be responsible for the compromised capacity to finely coordinate movements. It should be noted, however, that balance is not impaired following the isolated cooling of individual lower limb muscles [79]. Local cooling also failed to affect force sense [80] and the reflex responses of the hamstring muscles provoked by anterior tibial translation [81]. Jointly, these results suggest that the effects are likely dependent on the cooling protocol used and that not all sensory performances are affected to the same extent.

Overall, although studies examining the effects of cold exposure on strength performance are limited, available research indicates that specific strength tasks may be impaired already after relatively short stays at moderate cold ambient temperature, and that effects may be more pronounced when environmental stressors are combined. Therefore, when exercise is performed in the cold, besides warming up (described in detail in the next section), athletes should consider adequate clothing during the entire time of exposure (i.e., during exercise as well as during breaks) to prevent significant falls in skin temperature. Obviously, in some sports, athletes may be reluctant to wear excessive clothing, which may affect performance. Therefore, adequate clothing should not only be designed to keep athletes warm but also to allow maximal performance. Moreover, wherever possible, athletes may stay active during pauses in competitions or trainings to keep warm. Further studies are needed to evaluate if habituation to cold air would attenuate the detrimental effect of low ambient temperatures on strength performance.

### 2.3. Influence of Cold on “Warm-Ups” for Sport in Cold Weather

A well-known factor in sport performance is the use of a “warm-up” to enhance preparedness for a training bout/competition [82]. Most research regarding how a warm-up improves sport performance has been completed in temperate environments (indoors training facilities, laboratories, temperate climates) where more research on environmental conditions and warm-up has been called for [83]. However, cold weather sports have a number of risks relative to temperate environment sports [54], which increases the planning for training bouts and events (competitions) to ensure both good performance as well as athlete health [84]. However, there is limited research related to warm-up strategies in cold environments to enhance sport performance in cold weather sports. Thus, this section applies what is known about the strategies currently employed to enhance performance in cold weather sports.

Given that active and passive warming of the muscle is a key benefit of any warm-up for sport, it is important to first consider how cold environments might influence muscle temperature increases due to a warm-up, especially when the severity of cold air and wind can independently [85] and in combination accelerate the vasoconstrictor response, leading to decreased muscle temperature and decreased muscular performance in a dose-dependent manner [86]. In addition, rain and other forms of moisture can also significantly influence temperature of skin especially when clothing becomes wet, altering the insulative value of clothing [8]. In fact, where sweating due to heavy work outputs such as walking in deep snow [87] creates wet clothing, cooling of cutaneous and muscle tissue temperature results in thigh temperatures below 32 °C in exercising humans [88]. Thus, clothing can significantly alter the thermoregulation of an individual engaged in cold weather exercise, especially when the fabric becomes wet. For that reason, the following recommendations for quality of fabric for cold weather exercise have been provided: (1) fabrics must have good “ease of wicking action”; (2) fabrics should have a high “rate of drying”; (3) fabrics should have high capacitance for “moisture regain (the amount of moisture a material can absorb before it feels cold)” and (4) “the degree of insulation a material loses when it becomes wet” should be low [18]. Applied to a warm-up, evidence implies that cold weather athletes should wear protective clothing that does not retain moisture during warm-ups, have high insulative value, and be highly wickable, thereby maximizing the benefit that an active warm-up might have on muscle temperature. As previous research in warm environments has shown, exercise performance improves 2–5% per 1 °C increase in muscle temperature [89], thus the benefit of an active warm-up cannot be understated. Translated to extreme cold the percentage gain in exercise performance with increased muscle temperature might be less in cold environments, and will depend on the quality of the insulative clothing [18]. Nevertheless, cold weather athletes must employ warm-up strategies which not only maintain initial muscle temperature but also increase muscle temperature, if the full benefit of the warm-up is to be realized. Interestingly, merino-based wool fabric when worn against the skin has greater thermal insulation properties and water absorbency than synthetic underwear when participants exercised for 1 h at a sub-maximal exercise intensity in cool conditions (8 °C, RH = 55%) [90]. Furthermore, those authors found that shivering due to sweating affecting skin temperature was greater in the females compared with males [90]. This highlights that management of warm-ups and protective clothing in cold weather might differ between sexes. Nevertheless, if protective clothing is worn, the good news is that the time course of muscle temperature in temperate environments is rapid, increasing to 38–39 °C within 20 min of muscular work completed [91]. Yet, the muscle temperature needs to be maintained otherwise a rapid decrease in muscle temperature can also occur upon cessation of exercise [91], thus as indicated below, pre-meditated strategies to maintain the benefits of a warm-up in cold weather should employed and these warm-ups should perhaps differ for male and female athletes.

Despite the effect that cold weather can have on the efficacy of a warm-up, research on warm-ups in cold weather sports is scarce. In fact, the International Olympic Committee (IOC) indicates that more research in cold weather sports including understanding metabolic heat production and models of predicting body cooling in sports with differing velocities [43] should be undertaken. The IOC also cites the importance of warm-ups in sports where cold stress is a major factor, especially open-water swimming [43]. The focus on open-water swimming is due to the significant cooling effect open-water swimming can have on core temperature [62] even when protective clothing is worn [92]. The use of protective clothing in cold open-water swimming highlights the importance of protective clothing in any cold weather environment where inadequate clothing in cold weather leads to a number of cold-related physiological responses [93]. Shivering thermogenesis is the most vigorous response due to cold exposure, providing excellent defense of core temperature [94]. However, in the context of sport, shivering due to inadequate clothing pre competition can reduce glycogen availability, thereby impairing “exercise performance” [93]. Independent of shivering, exercise in −10 °C conditions may also alter fat metabolism, driven by skin temperature-dependent mechanisms [30]. This finding heightens the importance of protective clothing to maintain skin temperature [95] as part of both the warm-up and within winter sport competition. Sport specific exercise impairment has also been shown in cross country skiers where double poling performance was reduced at −14 °C when wearing a thin Nordic racing suit [69]. Those authors conclude that despite maintenance of core temperature, decreased muscular performance is associated with skin temperature decline (12.5 °C decrease over 70 min of exposure). Others have also shown that the endurance performance is compromised in temperatures < −4 °C when wearing a thin Nordic racing suit [36]. In both projects, authors indicate a future focus on protective clothing research to reduce cold stress in cross country skiers, especially in cold conditions.

Other research investigating warm-ups in winter sport on subsequent performance indicate that a warm-up can positively influence body temperature in recreational alpine skiers, although it was not clear whether the skiing was completed in subzero conditions [96]. More recently, elite junior alpine ski racers were found to benefit from a warm-up that combined both active and passive (heated lower body garment) methods on cycling performance in −7 °C [97]. The authors interestingly ensured that the heated lower body garment was used in the transition period from end of warm-up to start of the cycling, to ensure maintenance of muscle temperature. This methodological approach highlights a key aspect of pre-competition warm-up strategies, that the raised muscle temperature due to the warm-up be maintained via heated or insulative clothing in the transition to the competition start. In addition to muscle temperature-related effects on exercise performance, the positive effects that a warm-up can have on exercise-induced bronchoconstriction (EIB) in cold weather cross country skiing (−12 to 6 °C) has been shown [98]. In this regard, using a heat and moisture exchanger during the warm-up and during the period between warm-up and start of the competition might be an interesting approach deserving further scientific attention [99]. Put in the context of pre-competition time periods where athletes are waiting to start an event, lack of a warm-up can clearly alter muscle and respiratory function, whereas inadequate clothing likely influences force development, coordination and exercise intensity within a competition [93].

In summary, given our current understanding of warm-up strategies in cold weather sports, more research should investigate: (a) different types of insulative clothing to enhance muscle temperature; (b) the change in body temperature in the period of restitution (post warm-up to competition start); and (c) how high force development-short duration events such as alpine skiing to long duration-high metabolic output activities such as cross-country skiing differ in preparatory requirements. In each of these future areas of research, questions should answer how the magnitude of the cold exposure influences a warm-up and also consider the potential influence of sex on exercise performance in the cold.

## 3. Exercise in the Cold, Cold Exposure and Potential Health Issues

### 3.1. Short-Term Effects

Health has been defined as optimal physical, mental, and social states, where transient changes in any dimension of health can alter overall health [100]. Despite the positive influence exercise can have on health [101], environmental factors including cold weather [102] have known risks that are associated with exercise in the cold [103]. These acute changes in health due to cold weather can occur during and after exercise, resolving in the hours and days after an exercise bout and have been deemed cold-related injuries [84]. The primary systems that are affected by cold weather exercise include the respiratory [104], cardiovascular [34], musculoskeletal [105], and dermal [106] systems. Given that sports are a form of exercise, these acute changes to health due to cold weather exercise can be extended to participants in cold weather sports, where both training and competition can influence health status [14,106]. The following section identifies some of the key changes to health that can occur during and after training or competition in cold weather and provides some pragmatic recommendations to manage short-term health in athletes who compete in cold conditions or inclement weather.

Acute changes in respiratory health has been identified in a number of cold air high-ventilation type sports including Nordic sports [98], ice hockey [107], figure skating [108], speed skaters [109], and runners who train in the cold [110]. The underlying common respiratory health risk factor in these cold weather athletes [111,112] is the inspiration of large volumes of cold dry air in a workout or competition resulting in water loss from the airways, increasing the osmolality of the airway surface lining [113]. This results in the contraction of bronchial epithelial cells and release of a number of pro-inflammatory mediators, leading to airway smooth muscle constriction often described as EIB [114]. This acute airway constriction can be caused by intense exercise in as little as 8 min of all out exercise, where the EIB worsens with progressively colder temperatures (0 to −20 °C) [17]. The recovery of respiratory function to pre-exercise values is typically less than 30 min [115]; however, underlying airway hyperresponsiveness [116], ambient temperature post exercise [117], and individuals of a small stature [118] have all been shown to affect the severity of EIB [119] or length of the recovery period. Furthermore, respiratory symptoms are also consistently reported during the recovery period of a training bout [120] or after competition [121] in cold weather sports. Previous research has identified the most common symptomology as coughing, wheezing, chest tightness, and excessive mucus formation [122]. These symptoms are particularly prevalent after intense intervals [17], a cross country ski race [123], or other indoor cold air environments where ice-related sports compete [108,124] and seem not to be sex dependent [125].

Short-term changes in cardiopulmonary health due to cold weather sport are driven by thermoregulatory and autonomic nervous system factors, which causes alterations in cardiovascular dynamics. It is well understood that hypothermia (less than 35 °C core temperature) can lead to cardiac dysrhythmias including prolonged QR, QRS, and QT as well as ventricular and atrial fibrillations [84]. However, the degree of cold exposure which is related to the onset of a significant hypothermia-related cardiac event in terrestrial sports such as mountaineering and trail running [7] is unknown. More common alterations to cardiovascular health would be the influence of aqueous sports such as open water swimming [15] and winter swimming [126], where prolonged exposure can lead to hypothermia [127] and related cardiovascular alterations [15] that can be fatal [128].

The short-term effects of cold environments on musculoskeletal complaints or muscular injury in sport are extremely limited. The majority of evidence that indicates cold has an effect on the prevalence and severity of musculoskeletal complaints, is derived from occupational research in the cold [105]. Collectively, research from cold occupations indicate that compared with temperate climates where the same job is performed, workers in cold weather have >25% more complaints [129] and musculoskeletal complaints are correlated to the degree of cold exposure (both the severity and length of time spent in a cold air environment). Physiological aspects of cold stress research would support the idea that the risk of a serious musculoskeletal complaint is heightened via a number of temperature-dependent mechanisms related to muscle force output [66,89]. Although the causal links between muscle function and musculoskeletal complaint or injury are not clearly established, as leading authors in this field indicate, if “force production, velocity, power and manual dexterity” [130] (p. 177) are affected by cold, then the risk is likely greater in cold weather competitions [130]. Specifically, cold-related decrements in function might influence risk of injury because demands of high muscle force type events (such as alpine skiing) or long duration events where muscle fatigue is a factor (such as cross-country skiing or biathlon) might overwhelm the musculoskeletal system. Retrospective research in recreational alpine skiers would support the dose-dependent effect that cold air can have on knee injury risk [131]. Cold-related effects on neuromuscular function affecting joint function have also been shown in non-sport exercise conditions [66,132], supporting the general but limited evidence that cold may heighten injury risk in winter sport. Clearly, more research on the cold related effects of injury mechanisms and risk is required, examining whether winter sports have the same general risks associated with cold weather occupations and exercise [54].

Dermal conditions related to cold exposure have been categorized as freezing and non-freezing injuries [8], and the risk of cold weather training and competition on dermal and superficial areas of the body with high exposure to cold are well described [43,84]. Additional, less injurious complaints regarding dermal health of an inflammatory nature have been studied in skating sports [106], where body size and speed of the skater have been cited as increasing risk of dermatological conditions [84]. A key determinant of cold-related dermatological conditions is skin that undergoes rapid cooling especially in uncovered or thinly covered areas of the body, resolving typically in minutes to hours after cold exposure has ended [106]. It is also known that dermatological health is a function of event length or training time in combination with associated airspeed of respective winter sports [54] as well as wet clothing [95]. Thus, to ensure that short-term dermatological injury does not lead to chronic peripheral tissue injuries [133], proper protective clothing especially in high exposure areas of the body is critical [84]. Finally, it should be noted that a number of independent risk factors including sex and ethnicity and dependent risk factors such as nutrition and fatigue have been cited as increasing risk of both injurious and non-injurious dermatological conditions [84,93].

### 3.2. Long-Term Effects

In the general population, both low and high ambient temperature are associated with increased morbidity and mortality risk, but the majority of temperature-related mortality has been attributed to the contribution of cold [134]. Living in a cold environment has consistently been demonstrated to increase the risk of cardiovascular diseases, largely associated with elevated blood pressure values and thrombogenicity [135]. Furthermore, low ambient temperature may foster respiratory morbidity and in particular cold-induced pneumonia in the elderly [136]. However, also young and otherwise healthy people may be at increased risk to suffer from respiratory disorders when regularly performing heavy physical activity or exercise in the cold, and this risk is aggravated with pre-existing respiratory diseases, i.e., asthma, and under conditions of high air pollution [137].

Recreational and competitive outdoor sports activities in extreme environments, i.e., low ambient temperatures, are becoming more and more popular. In particular, winter sports such as downhill skiing, cross-country skiing, or ski mountaineering are commonly practiced in the cold. This is also true for those climbing to higher elevations as temperature is decreasing by about 6.5 °C per 1000 m gain in altitude [3]. Whereas regular physical activity is among the most important lifestyle components beneficially affecting cardiorespiratory fitness and healthy aging [138], performing it too often and too long in cold environment may represent one of the rare conditions where physical activity can induce detrimental effects [14,139]. Such adverse consequences pertain almost exclusively to the respiratory health of athletes [14,140] and usually develop in non-asthmatic subjects after long-term (months to years) intense endurance training [141]. Observations from Finland report a prevalence of cold-related symptoms of up to 50% in the general population [142]. Wilber and colleagues found an overall incidence of EIB in 23% of the 1998 U.S. Winter Olympic Team, but the incidence was highest (50%) in cross-country skiers (57% in females and 43% in males). Of note, EIB occurs in 70% to 90% of individuals suffering from asthma [143]. It is of utmost importance to deal timely and appropriately with this problem, as affected individuals may otherwise cease to participate in sports activities and no longer benefit from associated health benefits.

Whereas short-term exposure causes common cold air-induced rhinorrhea (“the skier’s nose”), sometimes accompanied by nasal congestion and sneezing [144], cold air-provoked long-term responses may include damage of the airway epithelium associated with changes in airway wall structure and function [145]. Beside some individual susceptibility, prolonged and repeated exercise-related hyperventilation of cold (and dry) air is considered the primary cause for development of EIB. It should be mentioned that cold air (close to zero °C) always means dry air. Thus, hyperpnea of cold air makes the airway surface fluid (ASF) evaporate faster than it can be restored, causing drying and hypertonicity of the ASF and cooling of the mucosa [145,146]. Consequences are vasoconstriction and reactive hyperemia, vascular leakage and edema, and the release of inflammatory mediators triggering smooth muscle constriction [147]. Understandably, this problem starts to become particularly relevant when nose-breathing changes to nose-and-mouth breathing at an exercise intensity level exceeding the associated minute ventilation of 30 L/min [145].

EIB may develop during and subside after an active sports career (at least in swimmers), but will likely remain during competitive activity [148], which emphasizes the importance of appropriate preventive and therapeutic measures. 

Several non-pharmacological approaches have been recommended to diminish the risk of EIB in the cold, including warm-up exercises for 10 to 15 min at moderate intensity with the intention to evoke a refractory period (where EIB symptoms are reduced) [149]. Others use measures for pre-warming and humidifying the inhaled air when exercising, e.g., by breathing through a face mask or scarf [150]. However, recent research demonstrated impaired maximal running performance and more pronounced physiological demands during submaximal exercise when wearing such heat-and-moisture-exchanging devices [151]. In addition, a diet rich in omega-3 polyunsaturated fatty acids [152] and/or low sodium intake (<1.5 g/d) [153] may provide some protective effects.

The use of short-acting beta-agonists (SABAs) represents the pharmacological preventive measure of choice [149,154,155]. Although the prophylactic use (applied 15 min before exercise start) of SABA is highly effective, its regular application may lead to tolerance, likely by downregulation beta-2 receptors on mast cells and smooth muscles of airway [156]. Thus, when applied on a daily basis, adding an inhaled corticosteroid (ICS) has been suggested to reduce the frequency of SABA application [155,157]. Alternatively, the use of a leukotriene receptor antagonist (LTRA) may be considered because tolerance does not develop [158]. With regard to current World Anti-Doping Agency (WADA) regulations, the following inhaled beta2-stimulants (at a maximal dosage) are allowed: salbutamol 1600 μg over 24 h; salmeterol 200 μg over 24 h; formoterol 54 μg over 24 h [155]. However, in certain cases (if medically justified) a therapeutic use exemption (TUE) can be applied also for the use of prohibited agents.

## 4. Conclusions and Practical Recommendations

Exercise is primarily pursued outside in a variety of environmental extremes, including exercising in cold air and in wet or freezing cold conditions. This topic affects many different sporting disciplines at many different levels of performance, which is why an evidence-based review of the current state of knowledge specific to preparation and risk reduction when exercising in the cold is a warranted addition to the existing literature. This review outlines the current literature available regarding the preparation of active individuals and athletes who specifically compete or exercise in cold environments and inclement weather. Practical recommendations related to both endurance and speed-power sports have been provided where appropriate, alongside considerations for management of both short-term and long-term health considerations. It needs to be considered that single specific sports may necessitate specific recommendations, which this review is not able to provide. Future research where individual sports performance in cold environments is the focus will better inform and further delineate individual athlete needs. We think that specific areas of interest include potential physiological gender differences, fitness status, the role of genetic predisposition to exercise performance in the cold, the value of habituation/acclimatization and personal experience to cold environments for enhanced performance, the efficacy of prophylactic pharmacological interventions and technological considerations relating to advancements in insulative clothing technology, and warm up strategies. In absence of more sport-specific research that delineates how cold environments may affect athlete performance and health, we feel some practical recommendations derived from present research are appropriate. These are found below, structured in a manner that allows useful reference for coaches, practitioners, and athletes.

### Practical Recommendations

1. Sport groupings: Specific groups can be categorized according to degree of exposure as well as by primary energy system requirement or fitness factor. In the context of this review the following suggested sports groups are provided to assist in the development of recommendations that could benefit sport performance and reduce health concerns.
  - Occasional sports exposed to colder air environments: These sports or activities are primarily non-winter sports but have occasional inclement weather situations that are terrestrial based, which might affect performance and health. These sports would include soccer, American football, cross country running, orienteering, cyclocross, ultra-endurance running/cycling events on trails, and marathon canoeing. In these sports/activities, the primary consideration would be to enhance performance in cool environments to extreme heat environments, however, due to climatic events or scheduling of competitions in cold weather regions of the world these sports might face unexpected cold exposure. For these sports, the recommendations, would be to increase the amount of protective clothing that is employed pre-competition, have additional clothing than normal for in competition, and ensure that the competitors maintain increased coverage of skin to reduce the convective, conductive, and radiation cooling. Given that these competitors are not habituated to cool or cold weather, preparation could include cold water immersion and increased preparation period in the new environment. Where appropriate education of coaches and athletes on the influence of cold environments on muscle temperature and cold related injury should be provided by competition organizers, and practitioners/support staff.
  - Cold weather and winter endurance sports: These sports or activities are primarily winter sports where competition occur in cold or sub-zero conditions. These sports would include cross country skiing, biathlon, speed skating disciplines, ski mountaineering, skijoring, as well as what are considered summer sports but also performed in the winter (fatbike cycling, winter road race, and trail running, winter triathlons). The primary consideration in these sports is that individuals are faced with training and competition-related exposure to cold environments that can range down to extreme −40 °C and these acute bouts of cold exposure can have specific short-term health effects that can lead to long-term health problems. Although it is unlikely that many competitions are held in environments < −20 °C, the risk to these individuals is real and therefore it is recommended that sport organizations and governing bodies provide clear evidence on the risks of training and competing in extreme cold environments. Furthermore, given the rate of cooling that can occur to both skin and muscle temperature, where prolonged exposure exacerbates the magnitude of cooling, more research should focus on the acute and performance-related effects in real-world sport environments. Additionally, the influence of sweating on accelerating tissue and core temperature highlights the importance of merino wool base layers, with subsequent layers that are insulative and highly breathable to water vapour. Clearly, some sports such as cross-country skiing, biathlon, and speed skating opt for lycra-based racing suits which offer little protection to cold air environments, and future research should liaise with clothing manufacturers to test improved race suit designs. Current respiratory research in the cold has also found that heat and moisture exchange devices are beneficial to performance and reduce the acute effects of cold air, thus increased awareness for competitors on these benefits should be employed.
  - Speed-power winter sports (with special consideration of other power sports with inclement weather): These sports or activities are primarily winter sports where competitions occur in cold and sub-zero conditions in protected and unprotected wind zones. This would include traditional winter sports such as alpine ski disciplines, freestyle ski disciplines, luge/bobsled and skeleton, ski jumping, short track speed skating, ice climbing. However, consideration could be given to downhill mountain biking, rock climbing, and track and field events where inclement weather might have significant effects on performance. The primary recommendations would focus on winter speed sports where, similarly to endurance winter sports, the total exposure to cold environments throughout the training year is high, affecting performance and health. Unlike endurance winter sports, the pattern of outdoor workouts would be more intermittent, where specific multiple high intensity bouts such as a ski run would be followed by rest or inactivity, affecting overall metabolic heat production. This would illuminate that in workout choices to maintain skin and muscle temperature are very important for these speed winter sports, especially with the additive effect that wind speed can have on cooling (where ambient wind as well as athlete velocity) affect the thermal balance. It is clear that speed sports utilize a different amount of protective clothing during work intervals, however, little is known regarding the rest periods that an alpine skier or luge athlete might have on thermoregulation and performance. Important recent information using non-human mannequins do show the importance of clothing to reduce shivering, sensations of cold and discomfort in the “sitting on chairlift” portion of alpine skiing. In this study, sweating was increased in the intense skiing part of the simulation in the double layer condition compared with single layer clothing condition, thus the fine balance between staying warm enough but not too warm is an area of continued research to be pursued [159]. In addition, the neuromuscular requirements for these sports are different than endurance sports and more research is required to understand the real-world influence of cold environments on neuromuscular and localized motor coordination. As indicated, some limited research has found that injury risk might be influenced by cold exposure in alpine skiing; however, joint cooling and sport-specific performance might be investigated more, to improve how we manage the performance of speed-power winter sport athletes. Of lesser widespread concern would also be the influence that cool environments might have on a track and field sprinter or a climber in a bouldering competition, where the outdoor conditions are relatively cool compared with normal competitions. Yet, in specific instances such as the Diamond League Track Series, athletes wear the same competition kit despite the widely varied competition environments of extreme heat (Monaco) to the drastically colder Lausanne Switzerland. This speaks to the importance of improved knowledge translation for coach education programs and practitioners working in these sports, to understand not only heat stress but cold stress performance implications.
  - Water based sports completed in the outdoors: These sports are characterized as being completed in water, where the influence of the aqueous environment can be up to 25 greater than terrestrial-based sports. These sports include open water swimming, swim portions of a triathlon, winter swimming, as well as white water kayaking where submersion is a significant risk. Interestingly, given the significant effect that cool and cold water can have on body temperature the evidence and guidance on safety for open water swimming is clear. The understanding of how cold water can also affect muscle performance, cognitive function and competition performance is also an area of contemporary research which has resulted in some clear changes to competition rules, including mandatory use of wetsuits at <18 °C water temperature and minimum water temperature based on sound science.
2. Generalized recommendations across all sporting groups:
  - There is paucity on data regarding resting or training in the cold (habituation) to improve performance in a cold environment. Therefore, no evidence-based recommendations can be given. Yet, research suggests that cold habituation reduces sympathetic nervous activation [94] with potential positive effects on performance (e.g., due to preserved blood flow). Furthermore, normal living in a cold environment may alter the metabolic response to acute cold exposure, thus, increased time periods for training in a cold environment prior to a competition might influence metabolism as well as alter the thermo behavioural aspects of exercise in the cold. In practical terms, borrowing from thermo behaviour evidence, allowing athletes to experiment with different combinations of clothing layering, could improve their preparedness for competition day. It is known, for example, that many cross-country skiers might change their base layer between their warm-up and competition start time, to ward off the effects of sweat accumulation due to the warm-up to maintain adequate skin temperature in race. Thus, pre-meditated strategies to manage clothing choices to enhance muscle temperature and allow for optimal thermal comfort should be employed in sport organizations and teams competing in the cold.
  - From a safety perspective, a greater understanding of how training status can affect the ability of an individual to compete at pace where heat production still matches heat loss is required. This same renewed research focus should also be extended to female and junior athletes, where known factors of anthropometry, muscular power, and overall cardiovascular fitness, known to influence heat production and thermal balance, differ from adult male competitors [160]. Given the evidence that exists in this domain, it is suggested that competition be modified in extreme cold conditions, and that junior categories and female categories be given greater consideration in these decisions. From a training perspective, it is certain that extreme cold conditions <−15 °C should necessitate cancellation of practice/training sessions or be moved indoors when possible. If athletes choose to exercise in the extreme cold, it is recommended that athletes not overdress, feeling thermal discomfort at the start of a workout, and wear breathable wicking clothing to reduce sweating-related complications. Special attention should be provided to extremities where additional battery-operated socks, boot covers, mitts, and mitt over covers are used to ensure protection of hands and feet. The head and face should be covered in breathable insulative headwear because exposed skin can induce a cascade of respiratory and autonomic responses that affect health status [161]. Guidance from occupational cold weather work in terms of cold exposure affects can be found here as reference point for sport strategies (occupational exposure and associated reactions).
  - From a nutritional perspective, glycogen stores should be adequately filled before competing in endurance sport in the cold (mainly in remote areas). Running out of energy induces performance loss. With decreased exercise intensity, heat production is reduced and since during exercise, peripheral vasoconstriction and related insulation does not become maximal, heat loss remains high and hypothermia may develop [6]. Conversely, under resting conditions, energy-depleted athletes still can retain heat because of the body’s ability to adapt the fuel source (switching to fatty acids) [20].
  - As pointed out above, EIB occurs commonly in athletes at all levels and may be especially provoked when exercising at cold ambient temperatures. Symptoms (such as dyspnea, cough, wheezing) are often mild or moderate and related performance impairment is not clearly attributable by the athlete to the existence of a respiratory disorder. Thus, appropriate screening for EIB is of utmost clinical importance, particularly in young athletes. For the diagnosis of EIB, an exercise challenge in dry air has been recommended by the American Thoracic Society (ATS) and European Respiratory Society (ERS). The appropriate exercise protocol to detect EIB consists of rapid increase in exercise intensity within about 2 to 4 min in order to provoke a high level of minute ventilation, i.e., about 20 times of FEV1 [162]. Following the exercise challenge, FEV1 is measured at 5, 10, 15, and 30 min. A commonly applied criterion for the percent fall in FEV1 during the 30 min post-exercise period (compared with pre-exercise FEV1) to diagnose EIB is ≥10%. For cold weather athletes, an exercise challenge performed at cold temperature, e.g., in a cold chamber, may be even more predictive for EIB diagnosis than the exercise challenge at ambient temperature [163], at least when compared with Eucapnic Voluntary Hyperpnea (EVH) [118].
